# Enamel remineralisation prospect of Moringa Oleifera hydrogel, eggshell hydrogel versus sodium fluoride varnish on artificially demineralised primary teeth: in vitro study

**DOI:** 10.2340/aos.v83.40623

**Published:** 2024-05-06

**Authors:** Mona Essam Eliwa, Yousra Mohamed, Ehsan Hossam

**Affiliations:** a Department of Operative Dentistry, Faculty of Dentistry, Ahram Canadian University, Giza, Egypt; b Pediatric Dentistry Department, Faculty of Dentistry, Ahram Canadian University, Giza, Egypt

**Keywords:** Moringa, eggshell, EDX, microhardness, remineralisation

## Abstract

**Purpose:**

The purpose of the present in vitro study is to investigate and compare the remineralising potential of Moringa Oleifera extract, eggshell, and sodium fluoride varnish on microhardness of artificially demineralised enamel of primary teeth with biomimetic minimally invasive approach following the world paradigm shift towards natural products in paediatric dentistry.

**Material and methods:**

Sample size included 44 primary molars. The mineral content and surface microhardness of all specimens were initially assessed using energy dispersive x-ray examination (EDX) and Vickers microhardness. The specimens were artificially demineralised for 96 h at a temperature of 37°C and then reassessed directly after demineralisation. The demineralised enamel specimens were randomly divided into four groups according to the remineralisation regimen utilised. Group 1: Artificial saliva (control); Group 2: Sodium fluoride varnish; Group 3: Eggshell hydrogel; and Group 4: Moringa Oleifera hydrogel. The specimens were stored for 8 days and then subsequently evaluated using EDX and microhardness assessment by Vickers microhardness test and scanning electron microscope (SEM).

**Results:**

Regarding the microhardness test, there was a significant difference between the Moringa Oleifera group and Eggshell group compared to fluoride varnish (*p* < 0.05). Regarding EDX analysis, there was a statistically significant difference (*p* < 0.05) between Moringa Oleifera group and Eggshell group compared to fluoride varnish as the highest values were for Moringa Oleifera and Eggshell. On the other hand, there was no statistically significant difference (*p* > 0.05) between Moringa Oleifera and Eggshell in both the measurements.

**Conclusion:**

Moringa Oleifera and Eggshell might be considered as a biomimetic natural material capable of guiding enamel tissue remineralisation in early carious lesion of primary teeth.

**Clinical relevance:**

This research demonstrated the capability for early enamel caries to be remineralised using novel materials with a naturally counterpart implicated in biomineralisation as proved to be more effective than traditionally used fluoride varnish in primary teeth.

## Introduction

The notion of minimum invasion dentistry (MID), a conservative philosophy that primarily highlights early diagnosis of carious lesions, remineralisation of tooth surfaces, and preservation of surrounding tooth structure, is currently gradually changing the paradigm of modern dental treatment. Dental caries prevalence is still very high in many developing countries which causes demineralisation of tooth structure. Demineralisation resulting from the loss of calcium and phosphate ions can be restored by using a non-invasive calcium phosphate delivery system.

Modern kinds of toothpaste, creams, and gels that include tricalcium phosphate or sodium fluoride are now regarded as the most efficient sources of the inorganic ions needed for enamel remineralisation. Over many years, the cornerstone of the non-invasive management of incipient carious lesions was fluoride. However, its ability to promote net remineralisation is limited mainly because its action is confined by the availability of calcium and phosphate ions. Unfortunately, fluoride lacks the ability to guide the formation of mineral crystals and fails to form oriented and ordered mineral crystals on the surface of enamel [1].

This prompted researchers to look for new remineralisation strategies that made use of various agents that have a track record of remineralisation. Presently, various organisations across the world, including World Health Organization (WHO), are promoting natural products for better health. In recent years, attention has been focussed on the use of natural products as they have the advantages of minimal side effects and being alcohol and/or sugar-free, which are the two most common ingredients found in over-the-counter products [2].

The latest trending remineralising agent, the eggshell powder (ESP), containing dentine-like substances is an example to the physical approach which plugs the open dentinal tubules with calcium and phosphate that precipitate and prevent the fluid diffusion through the tubules into the dentin sub-surface.

Eggshell is a treasury of minerals, mostly calcium carbonate, and is probably the greatest natural source of calcium. The eggshell also contains phosphorous, magnesium, strontium, and fluoride. Demineralisation resulting from the loss of calcium and phosphate ions can be re-maintained by a calcium phosphate delivery system, like in eggshell [3].

Moringa Oleifera has more calcium than milk, resulting in a high remineralisation effect of enamel and dentin [4]. The leaves and stems are known to have a large amount of calcium bound in calcium oxalate crystals. It is rich in potassium, iron, Vitamin C, and Vitamin A [5]. Moreover, occlusion of dentinal tubules by the formation of different-sized mineral particles was observed after the application of Moringa Oleifera [6]. Various animal studies have assessed the safety of Moringa Oleifera extracts and have demonstrated a high degree of safety [7]. Moringa Oleifera extracts have been previously evaluated on the basis of their relative polyphenol, flavonoid contents, and it was found that water extract of leaves exhibits the greatest antibacterial activity [8]. The primary teeth enamel is mineralised to a lesser amount as well as displays a superior diffusion coefficient and is hence more vulnerable to acid dissolution in contrast to the enamel of permanent dentition. Chemical demineralisation of teeth is induced by acidic attack through two primary ways: dietary acid consumption through food or drink/drugs, and microbial attack from bacteria present in the mouth. During an acidic attack, chemical dissolution of both the organic and inorganic matrix components occur. Early childhood caries affecting the deciduous teeth commonly appears as white spot lesions, prompting intensive preventive procedures that are capable of inducing remineralisation of such lesions in deciduous teeth [9]. To our knowledge, no research has been conducted comparing remineralisation effect of Moringa Oleifera extract hydrogel and sodium fluoride varnish with eggshell hydrogel on primary or permanent teeth. Therefore, this was the driving force behind our study.

The current study aimed to assess the remineralising potential of Moringa Oleifera hydrogel and eggshell hydrogel as, coping with the paradigm shift in oral healthcare toward natural remedies. The null hypothesis is Moringa Oleifera extract has a higher remineralisation potential than eggshell solution and sodium fluoride varnish.

## Materials and methods

This study was an experimental in vitro study which was approved by the Scientific Research Ethical Committee with number 16222, Faculty of Dentistry, Cairo University, Egypt. It was performed in the Departments of Paediatric Dentistry and Dental Public Health, and Dental Materials in the Faculty of Dentistry, Ahram Canadian University, and National Research Centre.

### Sample size calculation

The sample size was calculated depending on a previous study as a reference [10]. According to this study, the minimally accepted sample size was 11 per group, when the response within each subject group was normally distributed with a standard deviation of 0.78, the actual mean difference was 0.98, when the power was 80% and type I error probability was 0.05.

### Specimen selection and preparation

A total of 44 primary extracted teeth for shedding purposes were collected from the paediatric dentistry clinic, Ahram Canadian University Hospital after extraction due to shedding after signing a consent sheet at the paediatric dentistry department, Faculty of Oral and Dental Medicine, Ahram Canadian University in Egypt. Teeth were softly cleaned of residual debris and washed thoroughly under running water. Teeth were stored in sterile saline until use. Only teeth with intact enamel surface were included in this study. While teeth showing presence of cracks, fractures, white spots, decalcification, fluorosis, or developmental defects were excluded from the study. Specimens were embedded in self-cure acrylic resin and allowed to set to create blocks [7].

### Demineralisation of specimens

Teeth were sectioned at cementoenamel junction level. The cut crowns were covered with two layers of acid-resistant varnish leaving only a 4 × 4 mm facial window [7]. The Demineralising solution was composed of 50 mM acetic acid + 2.2 mM Ca(NO3)2.2H_2_O + 2.2 mM KH_2_PO_4_ + 0.1 ppm NaF. The pH of the solution was adjusted to 4.8. Teeth were immersed in the demineralising solution for 3 days. [11]. The demineralisation process was implemented at 37°C. Thereafter, samples were pressure flushed by water spray for 15 s followed by ultrasonic cleaning in deionised water 3 times (5 min per time) to terminate demineralisation [12].

### Remineralisation of specimens

#### Moringa Oleifera preparation

Lyophilised MOL extract was bought normally from the National Research Centre, Cairo, Egypt. Leaves were collected, washed, dried and ground. Dried powder was prepared through extraction with 80% ethyl alcohol. The combined ethanolic extract was evaporated till dryness at 45° C using a rotary evaporator under reduced pressure. The prepared mark was dissolved in water, frozen and lyophilised to obtain a lyophilised dry powder and prepared as a Nanogel hydrogel [7].

#### Sodium fluoride varnish

Vanish™ 5% Sodium Fluoride White Varnish sweetened by xylitol and contains a tri-calcium phosphate from 3M ESPE brand. It was stated to be containing 22,600 ppm fluoride.

#### Eggshell hydrogel preparation

The Chicken Egg Shell Powder was prepared by the calcination protocol given by World Property Intellectual Organization (WO/2004/105912: Method of producing ESP [11]. Chicken eggshell contains about 95% of calcium carbonate which on conversion to basic calcium oxide due to calcination, is responsible for an increase in alkalinity. Chicken eggs used were cleaned with distilled water and kept in hot boiling water for 10 min at 100°C to facilitate the removal of membranes. The eggshells were crushed and powdered to small particles with a sterile mortar and pestle. The tiny, crushed particles obtained were then kept in a muffle furnace (Neycraft Model JFF 2000) at 1200°C to make sure the resulting powder is pathogen free and then was formed into gel [13, 14].

ESP hydrogel dispersions were prepared through dispersing a suitable concentration of ESP in distilled water, followed by dissolving the gelling agent, sodium carboxymethyl cellulose, at a concentration of 5–10% under continuous stirring at 1,000 rpm using a magnetic stirrer. The formed hydrogel was kept at 8°C (in the refrigerator) for a period of 24 h before further use. 10% concentrations of ESP produced a paste of consistency that is appropriate for handling and application [15].

All operations were performed by a single operator. Following every remineralisation procedure, samples were incubated in an artificial saliva solution, (pH–7.2). This artificial saliva was changed every 24 h to maintain ionic balance and pH of solution. These procedures were performed once/day/for 8 days. Storage solution: artificial saliva composition consisted of: Na3PO4–3.9 mM, NaCl_2_–4.29 mM, KCl–17.98 mM, CaCl_2_–1.10 mM, MgCl_2_–0.08 mM, H_2_SO_4_–0.50 mM, NaHCO_3_–3.27 mM, Distilled water [16, 17].

**Group 1:** Each specimen was left untreated and stored in artificial saliva.

**Group 2**: The specimens were exposed to fluoride varnish to the enamel surfaces and left for 5 min, then delicately removed with cotton tips, and immersed in artificial saliva.

**Group 3**: Each specimen was treated with ESP gel for 5 min, then delicately removed with cotton tips and immersed in artificial saliva.

**Group 4:** Specimens were exposed to Moringa oleifera gel and left for 5 min, then delicately removed with cotton tips and immersed in artificial saliva [6]. Each group consisted of 11 teeth per group.

### Testing of samples

#### Microhardness assessment

Vicker’s micro hardness tester (Wilson Tukon 11102 micro hardness tester Buehler Germany) at a load of 100 gm. was used. The load was applied smoothly for 10 s and after load removal, two impression diagonals were measured, usually to the nearest 0.1-μm with a filar micrometre and were averaged. The Vickers hardness (HV) was calculated using: Microhardness value (MHV) = 1854.4 L/d2 (where the load L was in gf and the average diagonal d was in μm). The examiner took three indentations HV number at spacing of 100 microns and calculated the mean value. It was measured at baseline, after demineralisation and remineralisation.

#### Assessment of morphology

##### Energy dispersive X-ray examination

The enamel surfaces of specimens from each group were scanned by energy dispersive x-ray examination (EDX) Unit attached to SEM (Model Quanta 250 FEG – made in Holland). Elemental analysis of each surface was done. It was measured at baseline, after demineralisation and remineralisation.

##### Scanning electron microscope

SEM (Model Quanta 250 FEG – made in Holland) was used for the assessment of morphological changes at the enamel surfaces after remineralisation using enamel surface and cross-section micrographs were obtained in a back-scattered electron mode with 30 kV accelerate voltage magnification 200X and 4000X.

### Statistical analysis

Statistical analysis was performed with SPSS 20^1^® Graph Pad Prism,^2^® and Microsoft Excel2016.^3^ Exploration of the given data was performed using Shapiro-Wilk test and Kolmogorov-Smirnov test for normality, and revealed that all data originated from normal distribution (parametric data) in all groups. Accordingly comparison between different groups was performed by using One-Way ANOVA test followed by Tukey’s Post Hoc test for multiple comparisons.

## Results

### Microhardness testing

#### Comparison between different intervals (baseline, demineralisation and remineralisation)

**Figure 1:**
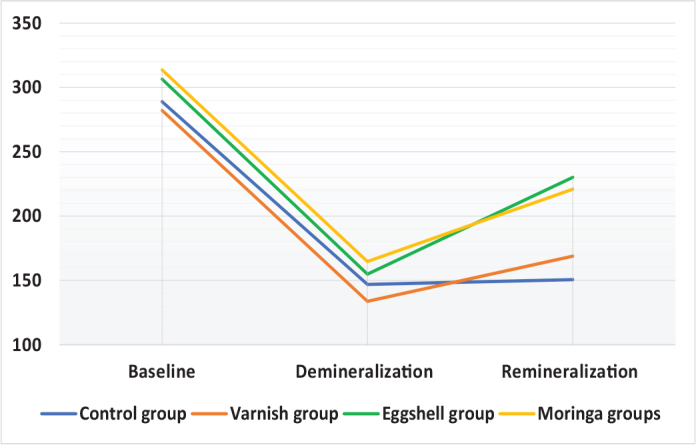
Line chart representing all groups at baseline, after demineralisation and after remineralisation. For group C and group D after remineralisation stage showed insignificant difference between each other.

**Figure 2:**
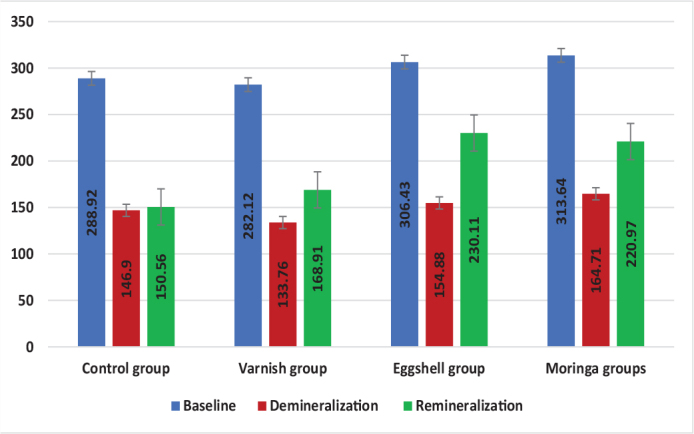
Bar chart representing all at baseline, after demineralisation and after remineralisation.

Comparison between different intervals revealed significant difference (*p* < 0.05) between all intervals in all groups, followed by Tukey’s Post Hoc test which revealed significant difference between means with different superscript letters as *p* < 0.05 (baseline and other intervals in control and varnish groups/significant difference between all intervals in Eggshell and Moringa groups, as presented in Table 1 and Figures 1 and 2.

**Table 1:** Mean and standard deviation at baseline, after demineralisation and after remineralisation in all groups and comparison between different intervals using Repetitive One-Way ANOVA test.

	Baseline		Demineralisation		Remineralisation		*P*
	M	SD	M	SD	M	SD	
**Control group**	288.92 ^aA^	15.52	146.90 ^bA^	73.52	150.56 ^bA^	52.62	<0.0001*
**Varnish group**	282.12 ^aA^	43.04	133.76 ^bA^	58.46	168.91 ^bAB^	59	<0.0001*
**Eggshell group**	306.43 ^aA^	24.37	154.88 ^bA^	71.09	230.11 ^cB^	66.93	<0.0001*
**Moringa groups**	313.64 ^aA^	23.22	164.71 ^bA^	60.7	220.97 ^cB^	43.12	<0.0001*
*P*	0.051		0.13		0.003*		

M: mean; SD: standard deviation. *Significant difference as *p* < 0.05.

Means with the same superscript letters (small per raw/capital per column) were insignificantly different as *p* > 0.05.

Means with different superscript letters (small per raw/capital per column) were significantly different as *p* < 0.05.

#### Comparison between different groups (control, varnish, eggshell and moringa)

Comparison between different groups performed revealed significant difference between them in remineralisation only as *p* < 0.05 , followed by Tukey’s Post Hoc test for multiple comparisons which revealed significant difference in means with different superscript letters as *p* < 0.05 (control group was significantly the lowest/varnish revealed insignificant difference with all other groups/eggshell group and moringa were significantly the highest with insignificant difference between them), as presented in Table 2 and Figure 2.

**Table 2:** Mean ± SD of elements Wt% for all groups at baseline stage.

Minerals	Control	Varnish	Eggshell	Moringa	*P* *
**C**	13.66 ± 1^A^	11.4 ± 3.19^A^	9.12 ± 2.67^A^	9.28 ± 2.04^A^	0.141^NS^
**O**	24.59 ± 1.57^A^	28.39 ± 4.76^A^	21.69 ± 3.31^A^	24.49 ± 2.69^A^	0.179^NS^
**P**	24.81 ± 2.08^A^	22.75 ± 1.43^A^	26.38 ± 2.32^A^	24.05 ± 1.65^A^	0.207^NS^
**Ca**	36.93 ± 4.08^A^	37.46 ± 3.14^A^	42.81 ± 3.65^A^	42.18 ± 8.56^A^	0.058^NS^

NS: non-significant (*p* > 0.05).

* Overall *p*-value of inter-group comparison.

### Energy dispersive X-ray examination

#### Effect of material type on the Wt% of elements within the same stage (intra-group comparison)

##### Baseline

**Figure 3:**
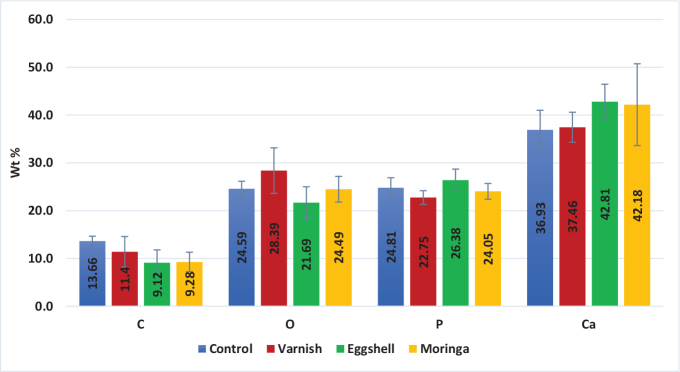
Bar chart representing mean and SD of elements Wt % at baseline stage for all groups.

**Phosphorus:** There was no statistically significant difference between the four groups (the means have the same superscript letter (A). The overall *p*-value was not statistically significant, as shown in Table 2 and Figure 3.

**Calcium:** There was no statistically significant difference between the four groups (the means have the same superscript letter (A). The overall *p*-value was not statistically significant, as shown in Table 2 and Figure 3.

##### Demineralisation

**Figure 4:**
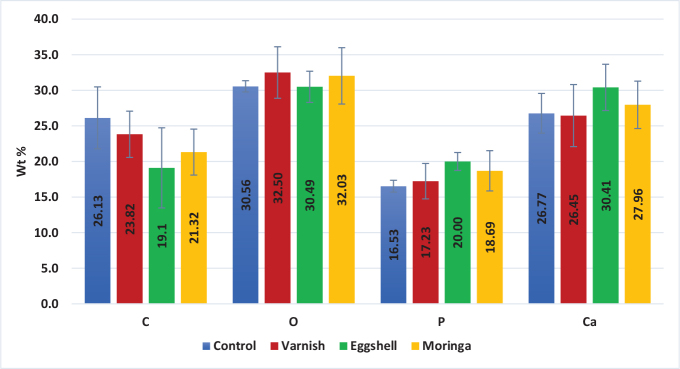
Bar chart representing mean and SD of elements Wt % at demineralisation stage for all groups.

**Phosphorus:** There was no statistically significant difference between the four groups. The overall *p*-value was not statistically significant as shown in Table 3 and Figure 4.

**Table 3:** Mean ± SD of elements Wt% for all groups at demineralisation stage.

	Control	Varnish	Eggshell	Moringa	P*
**C**	26.13 ± 4.35^A^	23.82 ± 3.25^A^	19.1 ± 5.63^A^	21.32 ± 3.23^A^	0.274^NS^
**O**	30.56 ± 0.79^A^	32.5 ± 3.62^A^	30.49 ± 2.19^A^	32.03 ± 3.96^A^	0.779^NS^
**P**	16.53 ± 0.83^A^	17.23 ± 2.49^A^	20 ± 1.26^A^	18.69 ± 2.83^A^	0.236^NS^
**Ca**	26.77 ± 2.79^A^	26.45 ± 4.36^A^	30.41 ± 3.25^A^	27.96 ± 3.33^A^	0.528^NS^

Capital letters for inter-group comparison (Control vs. Varnish vs. Eggshell vs. Moringa) and the means with different superscripts are statistically significant different at p ≤ 0.05

**Calcium:** There was no statistically significant difference between the four groups The overall *p*-value was not statistically significant as shown in Table 3 and Figure 4.

##### Remineralisation

**Phosphorus:** There was no statistically significant difference between the control and varnish groups (the means have the same superscript letter [B[), while there was a statistically significant difference between control group and the other two groups. The overall *p*-value was statistically significant.

**Figure 5:**
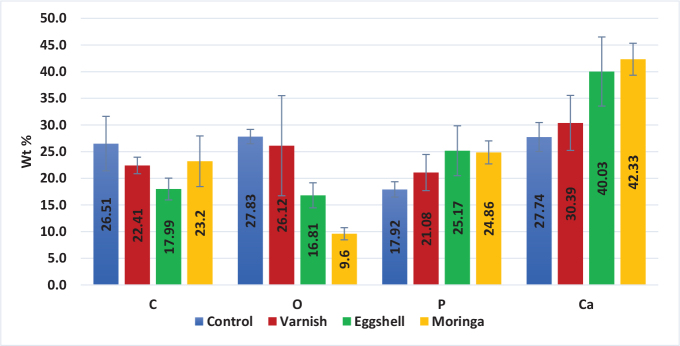
Bar chart representing mean and SD of elements Wt % at remineralisation stage for all groups.

**Calcium:** There was no statistically significant difference between the Moringa and Eggshell groups when compared to each other as different interventions(also, there was no statistically significant difference between the Control and Varnish groups in calcium content). The overall p-value was statistically significant as shown in Table 4. That reflects that there was an increase in Calcium&phosphorus Weight% in Eggshell group and Moringa Oleifera group more than varnish and control groups as shown in Table 4 and Figure 5.

**Table 4:** Mean ± SD of elements Wt% for all groups at remineralisation stage.

	Control	Varnish	Eggshell	Moringa	*P* *
**C**	26.51 ± 5.11^A^	22.41 ± 1.55^AB^	17.99 ± 2.04^B^	23.2 ± 4.75^A^	0.118^NS^
**O**	27.83 ± 1.34^A^	26.12 ± 9.39^A^	16.81 ± 2.35^AB^	9.6 ± 1.14^B^	0.005^S^
**P**	17.92 ± 1.44^B^	21.08 ± 3.39^A^	25.17 ± 4.68^A^	24.86 ± 2.16^A^	0.010^S^
**Ca**	27.74 ± 2.72^B^	30.39 ± 5.16^B^	40.03 ± 6.48^A^	42.33 ± 3^A^	0.001^S^

NS: non-significant (*p* > 0.05); S: significant (*p* > 0.05).

* Overall *p* value of inter-group comparison.

Capital letters for inter-group comparison (Control vs. Varnish vs. Eggshell vs. Moringa) and the means with different superscripts are statistically significant different at *p* ≤ 0.05.

### Assessment of morphology

**Figure 6:**
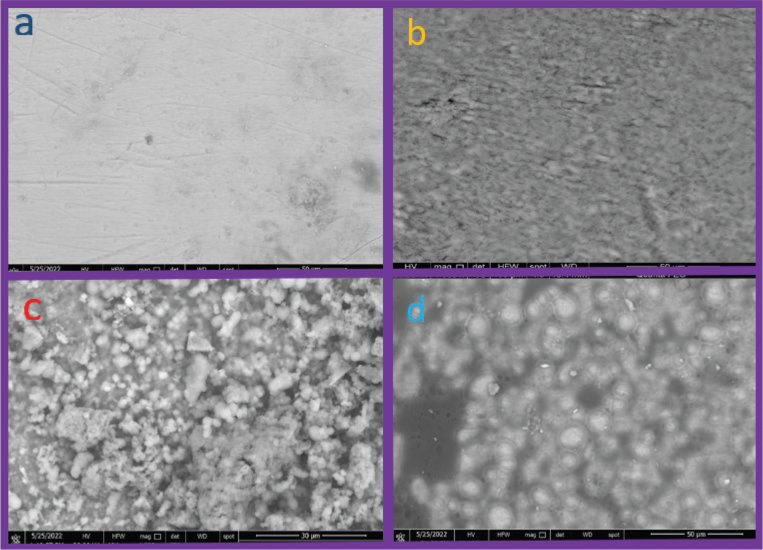
Group 1: control group (a): remineralisation. Group 2: fluoride varnish (b): after remineralisation. Group 3: Eggshell group (c): after remineralisation. Group 4: Moringa group (d): after remineralisation under magnification power 2000.

SEM images of control group showed that the surface of etched enamel surface had number of porous defects resembling demineralisation. Also, there was no typical integrity to the enamel surface (Figure 6, group 1). The surfaces of enamel treated with Eggshell group 3, remained relatively dense and intact compared with other groups filled with spheric inorganic minerals due to its calcium carbonate-based structure. The enamel surfaces in the group 4 treated with Moringa Olifera, showed a more accentuated continuous amorphous layer of mineral deposition with an irregular structureless appearance and an increase in the thickness of interrod regions and deepening of rod ends. Because of such well-organised crystal growth at the surfaces; widened inter-prismatic distance and micro porosities observed as in the control group were absent. Newly deposited rod-like structures found to be perpendicular to the enamel surface mimicking natural enamel rods (Figures 6c and d).

## Discussion

Biomimetic materials and crystal growth is a significant topic in material science and dentistry as a novel approach for treating dental caries. The era of preventive and minimally invasive dentistry clearly dictates the need for developing newer natural approaches to remineralise enamel caries lesions, especially in paediatric dentistry. In this study, we aimed to evaluate the power of natural remineralising agents as a conservative approach in caries control aiming to use a non-invasive approach rather than the traditional way (drill and fill). Moringa Oleifera hydrogel and Eggshell hydrogel were used in this study to test and analyse their potential for remineralisation. The quantitative evaluation of the enamel surface was carried out by measuring the surface microhardness. The microhardness test was chosen as a convenient method for a material having a non-homogenous, fine microstructure, and being prone to cracking like enamel surface microhardness indentation provides a relatively simple, reliable, non-destructive, and rapid method for evaluation of remineralisation changes in the tooth surface [18]. The topical application of eggshell on demineralised enamel, played a critical role in its remineralisation because of its high concentration of bioavailable Ca, and this explains the purpose behind its inclusion in this study [11].

Moringa Oleifera is a plant that is very rich in nutritional elements and has been used in treating many diseases. Its high content of calcium and potassium and many other natural proteins could be beneficial in presenting the required ingredients to remineralise initial caries lesions non-invasively to halt disease progression and aesthetics strength, and function of teeth. Developing a remineralisation protocol that inhibits demineralisation and encourages remineralisation remains a challenge in this field [19]. Thus, Moringa Oleifera hydrogel was selected in the present study.

In this study, the comparison between remineralisation efficacy of Eggshell hydrogel, Moringa Oleifera hydrogel and fluoride varnish was conducted as there was no sufficient data in the current literature comparing them together in the form of a hydrogel, documenting which one has a higher remineralising potential.

EDX analysis has been conducted in order to evaluate the elemental composition of the material. The results of atomic analysis by EDX showed a raise in calcium and phosphorous in samples treated with Eggshell hydrogel and Moringa Oleifera hydrogel compared to fluoride varnish group, which means that the enamel surface of the teeth regain their content of calcium and phosphorous. This may be attributed to Moringa high concentration of minerals [20]. These could play a role in the regulation of mineral deposition and guidance of enamel crystals formation. Although fluoride varnish is capable of deposition of minerals on enamel surface, still it’s not comparable to Eggshell hydrogel and Moringa Oleifera hydrogel. There was no significant difference between both agents. Following the application of remineralising agents on enamel caries-like lesions, it appears that mineral ions diffused into the superficial layer obstruct the surface porosities; further diffusion of minerals is limited after reaching a plateau [21]. This explains the lack of a significant difference in remineralisation of lesions following the use of Eggshell hydrogel and Moringa Oleifera hydrogel in our study which is confirmed by EDX.

Regarding the microhardness test, there was a statistically significant difference between the results of the Moringa Oleifera and Eggshell compared to fluoride varnish. This may be because the Moringa Oleifera leaves have been reported to be a rich source of β-carotene, protein, vitamin C, calcium and potassium, and have a high release of natural antioxidants [5]. These results were in agreement with Khalaf et al. [6], who revealed that the SEM analysis of enamel specimens after application of Moringa showed blockage of enamel prisms with the appearance of mineralised deposits along the porous defect. The way Moringa extract works is not clear, but could be explained through different possible mechanisms. Moringa increases the pH level in body fluids and therefore counteracts acidification which enhances remineralisation and is harmonised with the results of Gopalakrishnan et al. [20].

Regarding Eggshell remineralising effect, it showed a raise in microhardness test after remineralisation revealed by a statistically significant difference in the mean enamel surface microhardness between group 3 (treated with eggshell hydrogel) and group 1 (its control) and group 2 (fluoride varnish). This finding was in agreement with Mony et al. [10], who concluded that ESP has the potential to favour remineralisation due to its high pH and rich bioavailable calcium content. It was also supported by Haghoo et al. [22], who revealed that eggshell solution could be used as a remineralising agent for incipient enamel carious lesions, and it is as effective as nano hydroxyapatite for enamel remineralisation. Moreover, Yaberi et al. [23] reported that microhardness of the enamel significantly increased after treatment with Eggshell extract. Therefore, using these hydrogels biomimetic mineralisation model to regenerate tooth enamel is a promising method for the treatment of tooth enamel loss. It reflects the relevance of clinical application of Moringa and Eggshell hydrogel on primary teeth as natural varnish for preventing tooth decay and initial caries lesions, and arresting existing carious lesions. It is also very useful in special health care needs children liable to carious occurrence due to poor oral hygiene measures. Moreover, it can be used instead of fluoride varnish and foam application in the checkup visits for high caries index children.

## Conclusion

- Moringa Oleifera hydrogel and Eggshell hydrogel have the ability to increase surface microhardness of demineralised enamel even higher than fluoride varnish.
- Moringa Oleifera hydrogel has a remineralising power on caries like lesions when compared to another golden agent like fluoride, and could be used further in different vehicles as a natural antibacterial remineralising agent.
- Eggshell hydrogel has a promising future in treating initial enamel surfaces lesion due to its natural source of minerals and easy bioavailability. It may be used in the impending future for the remineralisation of early enamel caries.

## Recommendation

Further research is needed to study it as clinical trials and study précised chemical analysis of each agent with the amounts of active ingredients that would affect their influence on enamel remineralisation in different vehicles.

## Declarations

### Ethics approval and consent to participate

#### Ethics approval

The research proposal was revised and approved (approval number 16222) by the research ethics committee, faculty of dentistry, Cairo University, where the committee approved the procedures stated in the study.

## Consent to participate

The following study does not include any patient [human] data and not having direct involvement of human. They were just collected extracted teeth due to shedding purpose.

The study was conducted in accordance with the fundamental ethical principles and relevant guidelines and regulations in accordance with the Declaration of Helsinki.

## Consent for publication

Not applicable.

## Availability of Data and Material (ADM)

- The datasets used and analysed during the current study available from the corresponding author on reasonable request.

## Competing interest

Authors declare that there is no conflict of interest in this study.

## Author’s contribution

**Yousra Mohamed**: lecturer, Paediatric dentistry department, Faculty of Dentistry, Ahram Canadian University, Giza, Egypt.

**Mona Essam Eliwa**: Assistant Professor of Operative Dentistry, Faculty of Dentistry, Ahram Canadian University, Giza, Egypt.

**Ehsan Hossam El-Din Bayoumy:** Lecturer of Operative Dentistry, Faculty of Dentistry, Ahram Canadian University, Giza, Egypt.

## Footnotes

1 Statistical Package for Social Science, IBM, USA.

2 Graph Pad Technologies, USA.

3 Microsoft Co-operation, USA.
